# IntNetLncSim: an integrative network analysis method to infer human lncRNA functional similarity

**DOI:** 10.18632/oncotarget.10012

**Published:** 2016-06-14

**Authors:** Liang Cheng, Hongbo Shi, Zhenzhen Wang, Yang Hu, Haixiu Yang, Chen Zhou, Jie Sun, Meng Zhou

**Affiliations:** ^1^ College of Bioinformatics Science and Technology, Harbin Medical University, Harbin 150081, PR China; ^2^ School of Life Science and Technology, Harbin Institute of Technology, Harbin 150001, PR China

**Keywords:** long non-coding RNAs, lncRNA functional similarity, integrated network, lncRNA-disease associations

## Abstract

Increasing evidence indicated that long non-coding RNAs (lncRNAs) were involved in various biological processes and complex diseases by communicating with mRNAs/miRNAs each other. Exploiting interactions between lncRNAs and mRNA/miRNAs to lncRNA functional similarity (LFS) is an effective method to explore function of lncRNAs and predict novel lncRNA-disease associations. In this article, we proposed an integrative framework, IntNetLncSim, to infer LFS by modeling the information flow in an integrated network that comprises both lncRNA-related transcriptional and post-transcriptional information. The performance of IntNetLncSim was evaluated by investigating the relationship of LFS with the similarity of lncRNA-related mRNA sets (LmRSets) and miRNA sets (LmiRSets). As a result, LFS by IntNetLncSim was significant positively correlated with the LmRSet (Pearson correlation γ^2^=0.8424) and LmiRSet (Pearson correlation *γ*^2^=0.2601). Particularly, the performance of IntNetLncSim is superior to several previous methods. In the case of applying the LFS to identify novel lncRNA-disease relationships, we achieved an area under the ROC curve (0.7300) in experimentally verified lncRNA-disease associations based on leave-one-out cross-validation. Furthermore, highly-ranked lncRNA-disease associations confirmed by literature mining demonstrated the excellent performance of IntNetLncSim. Finally, a web-accessible system was provided for querying LFS and potential lncRNA-disease relationships: http://www.bio-bigdata.com/IntNetLncSim.

## INTRODUCTION

Recent large-scale genomic and transcriptomic analysis has shown that only less than 2% of genome sequence can encode protein, and functional non-coding transcripts constitute a large portion of the genome transcripts [1, 2]. Long non-coding RNAs (lncRNAs), a recently discovered class of non-coding RNAs, was arbitrarily defined as mRNA-like transcripts longer than 200 nucleotides that have no or little protein-coding capacity [3].

The accumulating evidence suggested that lncRNAs are a novel and crucial layer of gene regulation network, and play important roles in various biological processes, such as imprinting, developmental regulation, chromatin modification, transcriptional regulation, dosage compensation and so on [3–7]. The dysregulated lncRNA expression has also been observed and implicated in the development and progression of complex diseases [8–20]. Although tens of thousands of lncRNAs have been discovered and recorded in several public databases, such as GENCODE [21], NONCODE [22], LNCipedia [23], only a handful of lncRNAs were well-studied and characterized functionally. For example, only 182 functional lncRNAs were manually curated from existing literature in lncRNAdb [24].

It has shown to be an efficient way to infer potential function for novel genes by studying the functional similarity between genes with known functions or associated with specific diseases and that with unknown functions. Many methods have been developed to measure the functional similarity between protein-coding genes or miRNAs which accelerated functional analysis of protein-coding genes and miRNAs [25–28]. In consideration of the large number and limited knowledge of lncRNAs, it is urgent to develop novel methods to measure lncRNA functional similarity (LFS) for inferring lncRNA function and mining the associations between lncRNAs and diseases. Several efforts have been made to meet the urgent need in recent studies. For example, Sun and colleagues firstly introduced semantic similarity between lncRNAs-related diseases to calculate LFS (SemLncSim) which was used to predict disease-related lncRNAs [29]. SemLncSim was further improved by Chen *et al.* through considering lncRNA-disease associations and semantic similarity between diseases [30]. Another method, LFSCM, was proposed by Chen *et al.* to calculate LFS based on the lncRNA-related miRNA information [31].

Improved knowledge has suggested that lncRNAs were involved in diverse biological processes by negatively or positively regulating gene expression at both the post-transcriptional and transcriptional level [32]. For example, lncRNAs can function as key competing endogenous RNAs (ceRNAs) to communicate with mRNAs and regulate with each other by competing with common miRNAs at the post-transcriptional level [33, 34]. LncRNA also could negatively or positively regulate protein-coding gene expression in cis or trans at the transcriptional level. Thus, a more accurate measurement should take fully into account both lncRNA-related miRNAs/mRNAs and the functional communication among them. In this study, we developed an integrative framework, called IntNetLncSim, to infer human LFS by modeling the information flow in an integrated network that comprises both lncRNA-related transcriptional and post-transcriptional information. IntNetLncSim is freely accessible at (http://www.bio-bigdata.com/IntNetLncSim).

## RESULTS

### Performance evaluation of IntNetLncSim

**Figure 1:**
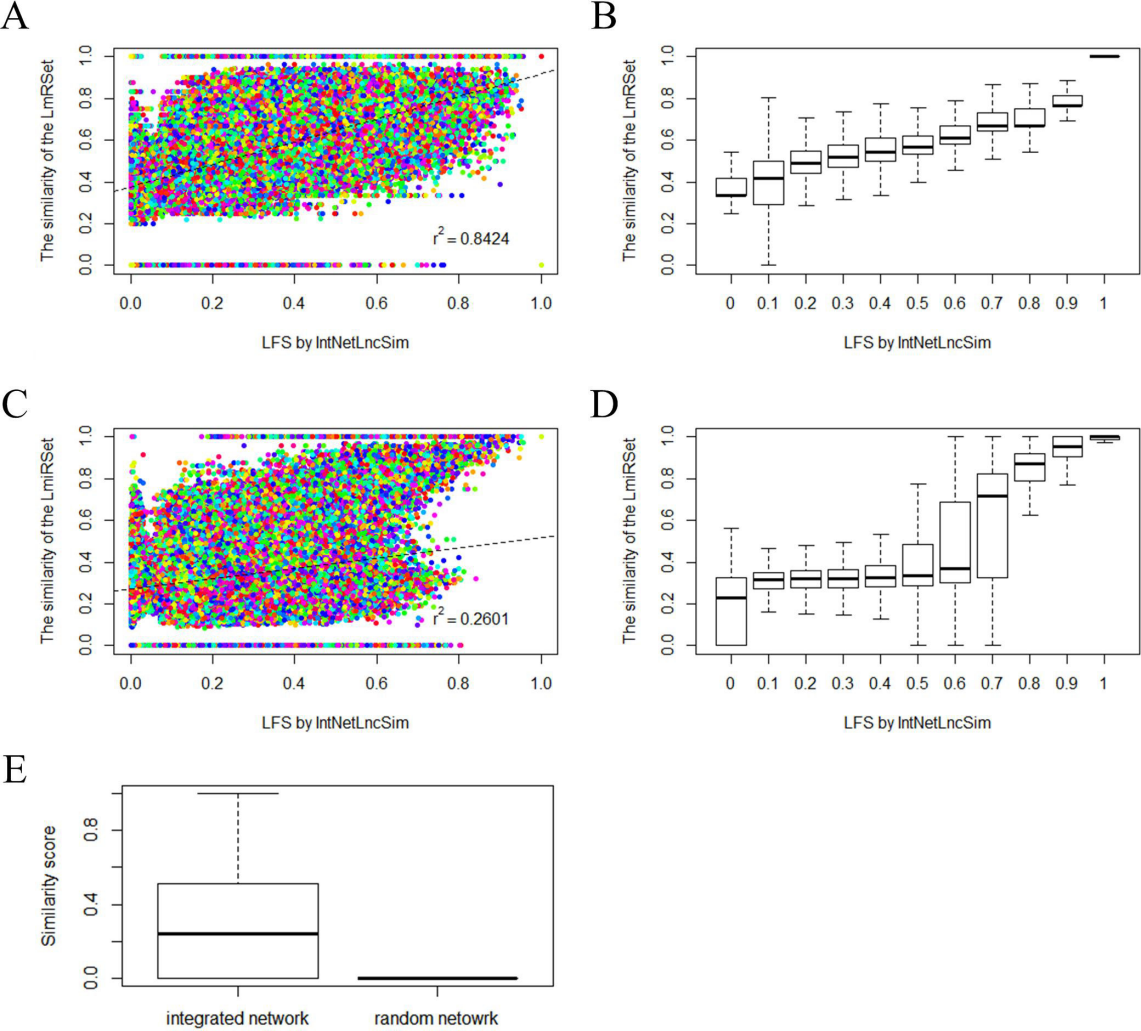
Performance evaluation of IntNetLncSim **A.** The distribution of the similarity of the LmRSet. A solid circle denotes the functional similarity of a pair of lncRNAs in the horizontal axis and the similarity of the LmRSet in the vertical axis. The dashed line is the linear regression line generated by the least squares of the data points. **B.** The distribution of the similarity of the LmRSet based on the grouped lncRNA pairs. **C.** The distribution of the similarity of the LmiRSet. **D.** The distribution of the similarity of the LmiRSet based on the grouped lncRNA pairs. **E.** The distribution of IntNetLncSim functional similarity scores of lncRNAs based on the integrated network and random network.

As mentioned above, lncRNA performed their function by negatively or positively regulating gene expression at both the post-transcriptional and transcriptional level. Therefore, it is expected that functionally related lncRNAs are often associated with functionally similar mRNAs or miRNAs. Therefore, we assessed relationships between IntNetLncSim functional similarity of lncRNAs and the similarity of the lncRNA-related mRNA sets (LmRSets) or miRNA sets (LmiRSets). In this study, functional similarity between mRNA sets was calculated by GsNetCom [35], which is a web-based toolkit to measure the functional association between two gene sets. In addition, functional similarity between miRNA sets was measured using Sun's method [25]. As a result, IntNetLncSim functional similarity of lncRNAs was significant positively correlated with the LmRSet (Pearson correlation *γ*^2^=0.8424, p=2.2e-16; Figure 1A) and LmiRSet (Pearson correlation *γ*^2^=0.2601, p=2.2e-16; Figure 1C). We further grouped lncRNA pairs into different groups according to LFS by a step of 0.1 and calculated the average LFS and the similarity of the LmRSet and LmiRSet. Then, the same correlation analysis was performed. As shown in Figure 1B and 1C, positive correlation between lncRNA functional similarity by our method and functional similarity of the LmRSet (Pearson correlation *γ*^2^=0.9753, p=3.299e-07; Figure 1B) and LmiRSet (Pearson correlation *γ*^2^=0.9448, p =1.181e-05; Figure 1D) was observed. Taken together, these results suggested that IntNetLncSim can reflect the correlations between LFS and that of LmRSet or LmiRSet.

To further verify the reliability of IntNetLncSim, a random network based on the topology of the integrated network was introduced. We first compared the relationships between IntNetLncSim functional similarity of lncRNAs based on the random network with the similarity of the LmRSet and LmiRSet, and then compared the LFS based on the random network with the similarity based on the integrated network. As expected, IntNetLncSim functional similarity of lncRNAs based on the random network was uncorrelated with the LmRSet (Pearson correlation *γ*^2^=8.267477e-05, p=0.717) and LmiRSet (Pearson correlation *γ*^2^=−0.0003, p=0.2117). In addition, the results in Figure 1E indicated that LFS based on the integrated network was significant difference with the similarity based on the random network (Pearson correlation *γ*^2^=0.0003, p=0.2117). The average LFS score based on the integrated network (0.3017728) was significantly higher than that based on the random network (8.540267e-07). Taken together, in comparison with random network, LFS based on the integrated network is more relevant with the similarity of the LmRSet and LmiRSet.

In order to assess the effects of mRNA and miRNAs in the integrated network, we ignored miRNA and mRNA, respectively. The correlation between LFS by ignoring miRNA and the similarity of the LmRSet is 0.7590, which is higher than that of IntNetLncSim (*γ*^2^=0.5385). However, the correlation between LFS by ignoring miRNA and the similarity of the LmiRSet (*γ*^2^=0.0467) is much lower than that of IntNetLncSim (*γ*^2^=0.2504). The correlation between LFS by ignoring mRNA and the similarity of the LmiRSet is 0.6735, which is higher than that of IntNetLncSim (*γ*^2^=0.2504). However, the correlation between LFS by ignoring mRNA and the similarity of LmRSet (*γ*^2^=0.0192) is much lower than that of IntNetLncSim (*γ*^2^=0.5385). Overall, the performance wasn't significantly affected after ignoring mRNA or miRNA. In comparison, the performance is more stable as using the integrated network. Because lncRNAs function at both the post-transcriptional and transcriptional level, the function of lncRNA could be reflected by both miRNA and mRNA. Therefore, the performance of the integrated network is more and stable.

### Comparisons with other existing similar methods

Because LFS of diverse lncRNA sets was calculated by different methods, the performance of IntNetLncSim should be compared with the performance of SemLncSim, LNCSIM, and LFCSM, respectively. For example, to compare the performance of IntNetLncSim and SemLncSim, common lncRNAs based on these two methods were extracted first. Then, the Pearson correlation between LFS of these common lncRNAs and the similarity of the LmRSet and LmiRSet could be calculated as following:

$$\rho_{\text{X},\text{Y}}=\frac{\text{cov}(X,Y)}{\sigma_{x}\cdot \sigma_{y}},$$ (1)

where X represents LFS using IntNetLncSim or SemLncSim, Y is the similarity of the LmRSet or LmiRSet, σ_x_ and σ_y_ are the variance of X and Y, respectively, and cov(X,Y) represents covariance between X and Y. Finally, the performance of IntNetLncSim and SemLncSim can be reflected by this correlation.

**Figure 2:**
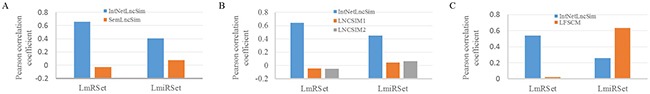
The comparison of IntNetLncSim with previous similar methods **A.** The correlation between LFS by IntNetLncSim and SemLncSim and the similarity of LmRSet and LmiRSet. **B.** The correlation between LFS by IntNetLncSim and LNCSIM and the similarity of LmRSet and LmiRSet. **C.** The correlation between LFS by IntNetLncSim and LFSCM and the similarity of LmRSet and LmiRSet.

SemLncSim was the first method to compute the similarity between lncRNAs. The correlations between functional similarity of lncRNAs and the similarity of the LmRSet and LmiRSet were shown in the Figure 2A. Obviously, the correlation between LFS by IntNetLncSim and the similarity of the LmRSet (*γ*^2^=0.6596, p=2.2e-16) is significantly higher than that of SemLncSim (Pearson correlation *γ*^2^=−0.0293, p=0.259). Although correlation (0.0737) seems to be improved slightly between LFS by SemLncSim and the similarity of the LmiRSet (Pearson correlation *γ*^2^=0.0737), the significant level of this correlation was also very low (p=0.1364). In comparison with SemLncSim, the correlations between LFS by IntNetLncSim and the similarity of the LmiRSet were much higher (Pearson correlation *γ*^2^=0.4064, p=2.2e-16). These results showed that LFS by IntNetLncSim is much more relevant with the similarity of the LmRSet and LmiRSet than LFS by SemLncSim.

LNCSIM was another method to calculate the similarity between lncRNAs which utilized the semantic similarity between diseases. LNCSIM1 and LNCSIM2 are two types of LNCSIM based on Resnik's [36] and Wang's method [37], respectively. After combining with IntNetLncSim, 55 common lncRNAs and 29 common lncRNAs that can regulate mRNAs and miRNAs were obtained, respectively. The correlations between functional similarity of these common lncRNAs and the similarity of the LmRSet and LmiRSet were shown in the Figure 2B. As a result, the performance of LNCSIM1 and LNCSIM2 appeared to be roughly the same. For example, the correlation between LFS by LNCSIM1 and the similarity of the LmRSet is −0.0436 (p=0.0931), and that of LNCSIM2 is −0.0467 (p=0.0722). In contrast, IntNetLncSim achieved a better performance. For example, the correlation between LFS by IntNetLncSim and the LmRSet is 0.6445 (p=2.2e-16). These results showed that the performance of IntNetLncSim is much better than LNCSIM.

LFSCM measures LFS between lncRNAs based on the miRNA information of lncRNAs. These interactions are part of the lncRNA regulatory network. Thus, lncRNAs in LFCSM are contained in IntNetLncSim. As shown in Figure 2C, the correlations between LFS by LFSCM and the similarity of the LmiRSet is 0.6330, which is higher than that of IntNetLncSim (*γ*^2^=0.2626). However, the correlations between LFS by LFSCM and the similarity of LmRSet (*γ*^2^=0.0244) is significantly lower than that of IntNetLncSim (*γ*^2^=0.5400) (Figure 2C). If only considering the correlation based on the similarity of the LmiRSet, the performances of LFSCM and IntNetLncSim were both very well. After introducing correlation based on the similarity of the LmRSet, the advantage of IntNetLncSim was obvious. These results showed that IntNetLncSim was more comprehensive and stable.

### lncRNA functional similarity network (LFSN)

We calculated similarity scores for all the pairs of lncRNAs in our integrated network by IntNetLncSim. Then, we got the z-score of these similarity scores. As a result, one-sided P-value was accessed for each similarity score. These LFS scores with P-values were used to construct LFSN (http://www.bio-bigdata.com/IntNetLncSim), which was utilized to predict novel associations between lncRNAs and diseases in the next section.

### Case studies

**Figure 3:**
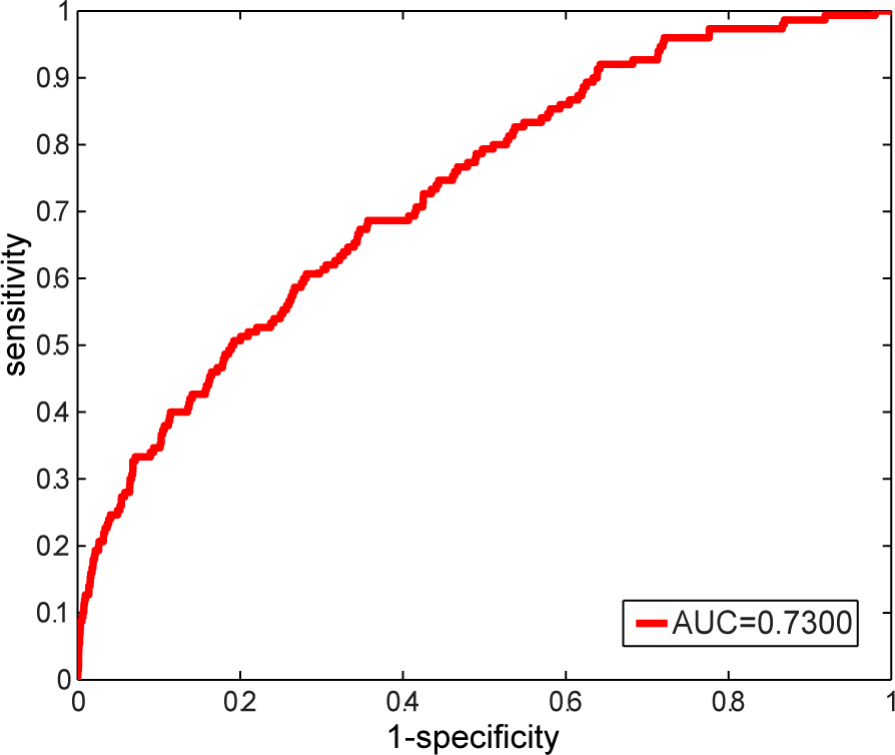
ROC curve and AUC value of our method based on leave-one-out cross validation on 150 known experimentally verified lncRNA-disease associations

By applying the above constructed LFSN, novel candidate disease-related lncRNAs were predicted based on random walk with restart (RWR) algorithm (see Materials and Methods). To evaluate the performance of the LFSN, leave-one-out cross validation of 150 known experimentally confirmed lncRNA-disease associations, including 40 diseases with at least two lncRNAs, was used for this assessment. For a disease *d* of interest, each known lncRNA associated with disease *d* was left out as the testing case, and the remaining known disease *d*-related lncRNAs were used as seed nodes. All the lncRNAs except the known disease *d*-related lncRNAs were considered as candidate lncRNAs. We then examined how well the testing lncRNA ranked relative to the candidate lncRNAs. If the ranking of this testing lncRNA exceeded a given cutoff, we regarded this lncRNA-disease association as successfully predicted. As a result, an area under the ROC curve (AUC) of 0.7300 was achieved (Figure 3), which demonstrated that our constructed LFSN was effective in recovering known experimentally confirmed disease-related lncRNAs.

To further indicate the application of our constructed LFSN in identifying novel disease-related lncRNAs, case studies of liver cancer and breast cancer were examined. For a given disease, the known disease-related lncRNAs were served as seed lncRNAs, and all the non-seed lncRNAs were ranked based on RWR algorithm. The top 20 lncRNAs in the ranked list were investigated. We manually checked these lncRNA-disease associations in the published literature and the results were shown in Table 1. Two and three of the top 20 predicted lncRNAs were validated in liver cancer and breast cancer, respectively, and most of them had high ranks in the predicted lncRNA lists. For example, expression quantitative trait loci in *ZNRD1-AS1* were recently found to affect both HBV infection and liver cancer development [38]. High expression of *NEAT1* in patients with breast cancer was reported to be correlated with poor survival [39]. All these results indicated that our constructed LFSN was effective in identifying novel disease-related lncRNAs, and the LFS method we proposed was reliable.

**Table 1:** The novel lncRNA-disease associations confirmed by literature mining

lncRNA name	Ranking	References
**Liver cancer**		
ZNRD1-AS1	10	[38]
ZNF718	20	[52]
**Breast cancer**		
SNHG1	8	[53]
NEAT1	9	[39]
SEC22B	12	[54]

### System design and implementation

**Figure 4:**
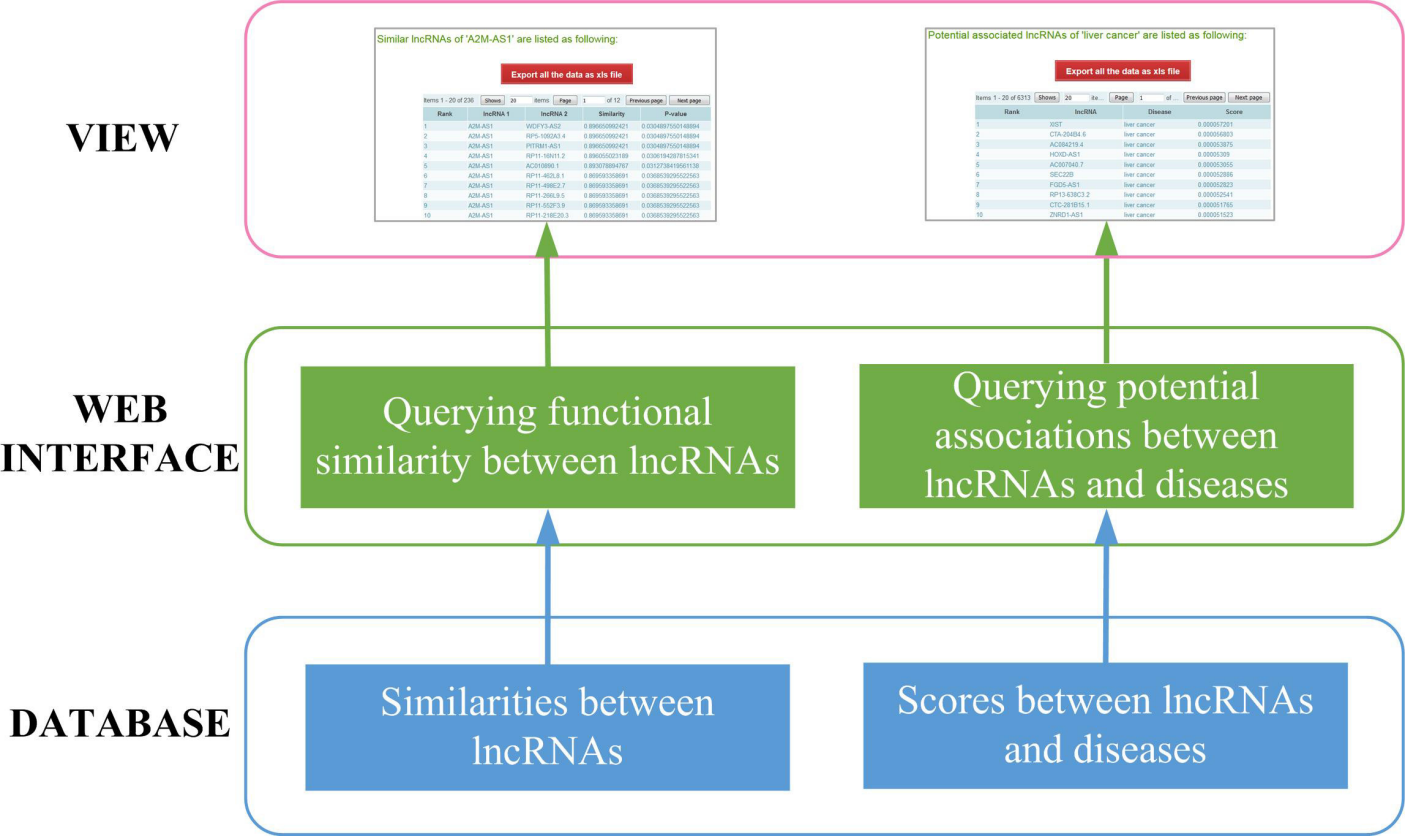
System overview

In order to facilitate querying lncRNA functional similarities and potential associations between lncRNAs and diseases, a web-based system was designed and implemented. The system was implemented on a JavaEE framework and run on our web server (http://www.bio-bigdata.com/IntNetLncSim). The three-layer architecture involving DATABASE, WEB INTERFACE, and VIEW layer is shown in Figure 4.

## DISCUSSION

The importance of the function of non-coding RNA had been reflected in the previous research. Unfortunately, functional inferring of non-coding RNA is not easy in comparison with those of coding RNA. LncRNA is a new type of non-coding RNA that contribute to the largest number of RNAs in human, so it is urgent to develop novel methods for infering function of lncRNA. Recently, the function similarity of lncRNAs was proved that can be used to find potential function of lncRNAs [29]. In this study, we devised a new method, IntNetLncSim, for improving the performance of calculating the LFS by the integrated network. And then, the method was utilized to construct LFSN for predicting novel associations between lncRNAs and diseases. Furthermore, a web interface (http://www.bio-bigdata.com/IntNetLncSim) has been designed for accessing LFS and associations between lncRNAs and diseases.

IntNetLncSim is based on an integrated network involving lncRNA regulatory network, miRNA-mRNA interaction network, and mRNA-mRNA interaction network. In comparison with several previous methods, the integrated network covered much more lncRNAs (Table 2). Moreover, the performance of IntNetLncSim was proven to be very reliable and stable in the correlation with the similarity of the LmRSet and LmiRSet.

**Table 2:** The number of lncRNAs in SemLncSim, LNCSIM, LFCSM and IntNetLncSim, respectively

Method	The number of lncRNAs	Data Source
SemLncSim	129	LncRNADisease
LNCSIM	104	LncRNADisease
(LNCSIM1, LNCSIM2)		
LFCSM	1114	starBase
IntNetLncSim	6314	starBase

LFSN was constructed based on the functional similarity between lncRNAs by IntNetLncSim. The performance of LFSN was proven to be reliable for recovering experimentally verified lncRNA-disease associations from LncRNADisease by leave-one-out cross validation. Then, the LFSN was applied to predict novel lncRNA-disease associations not in LncRNADisease. Five predicted associations between lncRNAs and two kinds of important cancer (liver cancer and breast cancer) were validated from the latest researches. This means that the LFSN could be exploited to predict novel relationships between lncRNAs and diseases.

It should be noted that IntNetLncSim relied on our integrated network. According to Figure 1E, the result of random work could be affected by a large amount of missing interactions among mRNAs, miRNAs, and lncRNAs. Therefore, the performance of IntNetLncSim may be improved by the exposal of newly interactions among mRNAs, miRNAs, and lncRNAs.

## MATERIALS AND METHODS

### Data source

#### Human mRNA-lncRNA and miRNA-lncRNA interaction data sets

The mRNA-lncRNA interaction and miRNA-lncRNA interaction data sets were downloaded from starBase v2.0 database [40] in October 2015, which provided experimentally confirmed mRNA-lncRNA and miRNA-lncRNA interactions based on large scale CLIP-Seq data. Currently, a total of 17,609 mRNA-lncRNA interactions between 33 mRNAs and 6,238 lncRNAs and 10,212 interactions between 277 miRNAs and 1,127 lncRNAs were included in this study. These miRNA-lncRNA interaction and miRNA-lncRNA interaction data sets were integrated to form a lncRNA regulatory network.

#### Human mRNA-mRNA interaction data

The mRNA-mRNA interaction dataset was downloaded from Human Protein Reference Database (HPRD) [41]. The HPRD is a resource for experimentally derived information about the human protein–protein interactions, and proteins in HPRD were mapped to mRNAs. After getting rid of duplicate interactions, 39,239 interactions between 9,616 mRNAs were obtained and formed an mRNA-mRNA interaction network.

#### Human miRNA-mRNA interaction data

The miRNA-mRNA interaction dataset was retrieved from three widely used and experimentally confirmed miRNA-target databases: TarBase (version 6.0) [42], miRTarBase (version 4.5) [43] and miRecords (version 4) [44]. These three databases were merged and the name of mature miRNAs were unified using miRBase (Release 21) [45]. Finally, 37,832 targeting pairs involving 558 miRNAs and 12,370 target genes were obtained to form a miRNA-mRNA interaction network.

#### Human lncRNA-disease association data

The human lncRNA-disease association data was incorporated into the LFSN to predict disease-related lncRNA. These associations were accessed from LncRNADisease [46], which is a resource that curated the experimentally supported disease-lncRNA association data. After discarding disease terminologies not in Disease Ontology (DO) [47] and getting rid of duplicate associations, 189 associations between 79 diseases and 60 lncRNAs were obtained.

### Methods

#### Method for calculating lncRNA functional similarity

**Figure 5:**
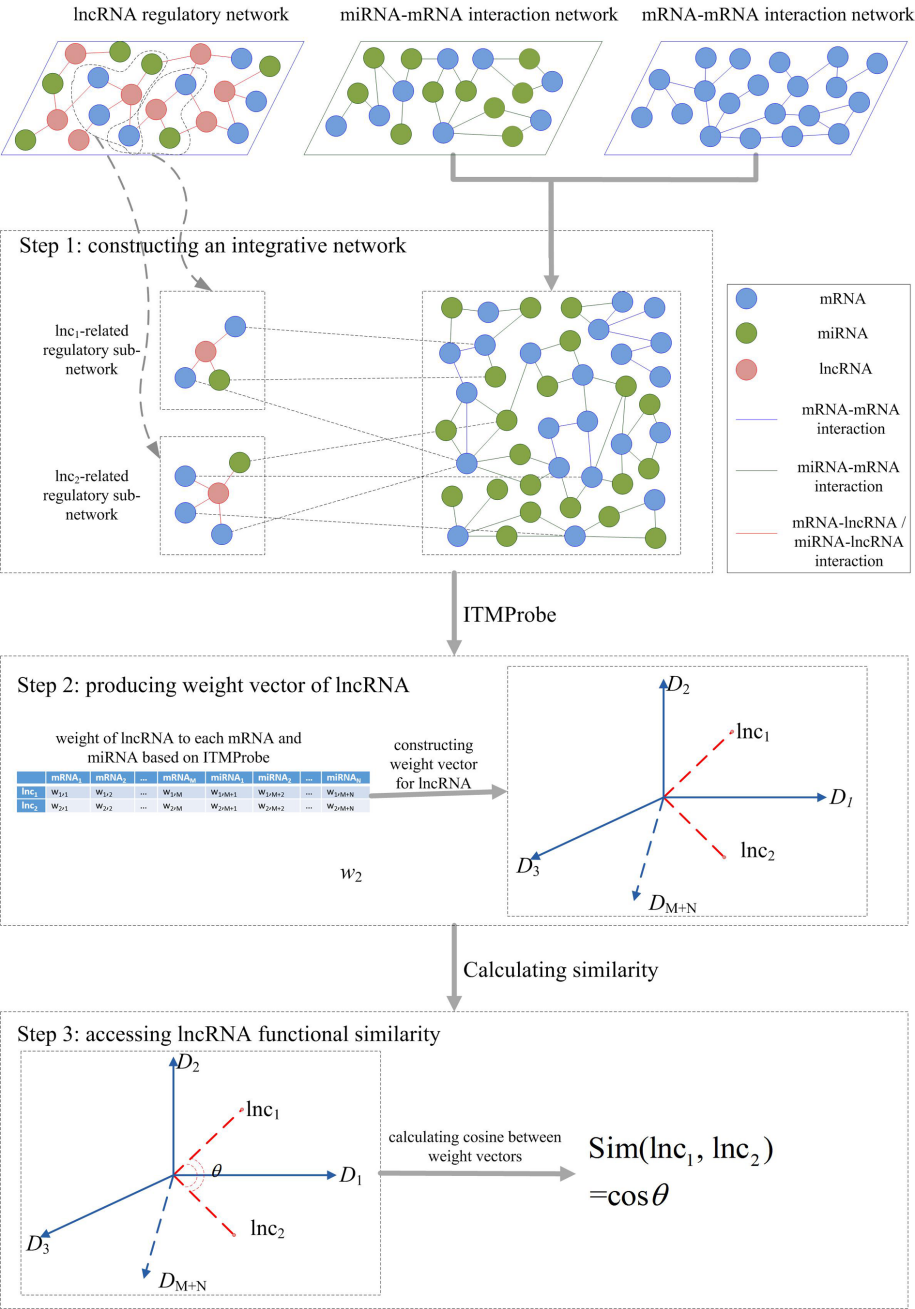
Overview of IntNetLncSim demonstrating the basic ideas of measuring lncRNAs functional similarity

In this study, we presented an integrative framework, IntNetLncSim, to measure the functional similarity of lncRNAs by modelling the information flow in an integrated network that comprises both lncRNA-related transcriptional and post-transcriptional information. A schematic representation of the IntNetLncSim method is shown in Figure 5. Initially, *lnc_1_* and *lnc_2_* are two lncRNAs. First, an integrative network was constructed based on lncRNA-regulatory network, mRNA-mRNA interaction network, and miRNA-mRNA interaction network. Then, ITM Probe [48] was applied for assigning a weight to each mRNA and miRNA for lncRNA by the integrative network. As a result, each lncRNA could be represented as a vector of these weights whose dimension equals the number of mRNAs and miRNAs in the network. Finally, the cosine similarity between vectors after using ITM Probe, which was implemented for calculating disease similarity in recent research [49], was exploited to calculate similarity of lncRNAs.

ITM Probe [48] is a tool for analyzing information flow in the network based on random walk with damping. Three models including absorbing, emitting, and channel were implemented in ITM Probe. Given a set of information sinks, the absorbing mode returns for any network node the likelihood of a random walk starting at that node to terminate at sinks. The emitting mode returns for each network node the expected number of visits to that node by a random walk starting at information sources. However, the directed flow from origins to destinations was induced via a potential function that was heuristic. Fortunately, channel model extends the absorbing model and emitting mode for directed information flow. According to these three models, all the nodes in the network were classified as boundary nodes and transient nodes. The boundary nodes contain source nodes that the random walk starts from and sink nodes that the random walk dissipates or ends at. And the transient nodes are neither source nodes nor sink nodes. After assigning boundary nodes and transient nodes, weights between these nodes could be outputted by the ITM Probe.

In this study, channel model in ITM Probe was applied to the integrative network. In this network, lncRNAs are not connected to each other, but they are linked to the mRNAs or miRNAs that are associated with them. The mRNAs and miRNAs are connected based on their curated interactions. Therefore, lncRNAs in the network were specified as boundary nodes, and all the mRNAs and miRNAs were specified as transient nodes, and with a damping factor of 0.85 according to previous research [50]. To assign a weight to each transient node for lncRNA, we consider a given lncRNA as source node and sink node in the information flow. Assuming *N* mRNAs and *M* miRNAs exist in the integrative network, each lncRNA can be represented as (*N*+*M*)-dimension vector based on the ITM Probe. For a given lncRNA *lnc_1_*, the weight vector can be described as

$$WV_{\text{ln }c_{1}}=\{w_{1,1},w_{1,2},\ldots ,w_{1,i},\ldots ,w_{1,N+M}\},$$ (2)

where $WV_{\text{ln}c_{1}}$ means a weight vector of *lnc_l_*, and *w*_l,i_ represents the weight score of *lnc_1_* on the *i*th dimension. Then, we modeled the functional similarity between lncRNA *lnc_1_* and *lnc_2_* by the cosine of their vectors as following:

$$\text{Sim}\left(\text{ln }c_{1},\text{ln }c_{2}\right)=\frac{\sum_{i=1}^{N+M}w_{1,i}\cdot w_{2,i}}{\sqrt{\sum_{i=1}^{N+M}w_{1,i}}2\sqrt{\sum_{j=1}^{N+M}w_{2,j}}\;2}.$$ (3)

#### Method for predicting disease-related lncRNAs

Disease-related lncRNAs were predicted using RWR analysis [51]. RWR is a global network ranking algorithm. The random walker starts on one or several seed nodes and then randomly transits to neighboring nodes considering the probabilities of the edges between the two nodes. The random walker can also return to the seed node, whose probability is supposed as *γ*, and then RWR algorithm can be defined as follows:

$${\text{P}}_{t+1}=(1-\gamma )\text{A}{\text{P}}_{t}+\gamma {\text{P}}_{0}.$$ (4)

Here, *P_0_* denotes the initial probability vector. *P_t_* is a vector in which the *i*th element indicates the probability of finding the walker at node *i* at step t. *A* is the column-normalized adjacency matrix of the LFSN. The algorithm was performed until the probability of all the nodes become stable, and was defined as P_∞_. This can be measured by the difference between *P_t_* and *P_t+1_* (measured by the L_1_ norm) falling below 10^−10^.

**Figure 6:**
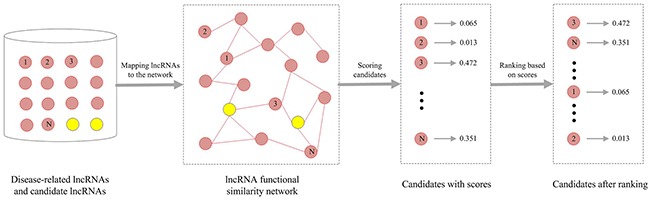
Flowchart of predicting disease-related lncRNAs

In this study, we predicted disease-related lncRNAs based on the constructed LFSN. The workflow was shown in Figure 6. For a given disease, the known disease-related lncRNAs were considered as seed nodes, while the rest lncRNAs were regarded as candidate lncRNAs. The seed nodes were mapped to the LFSN and a lncRNA rank list was then obtained using RWR algorithm. Each lncRNA was assigned a probability value in the above ranked list. The top ranked lncRNAs would have higher probability to be associated with a given disease.
